# Metagenomes of a Freshwater Charavirus from British Columbia Provide a Window into Ancient Lineages of Viruses

**DOI:** 10.3390/v11030299

**Published:** 2019-03-25

**Authors:** Marli Vlok, Adrian J. Gibbs, Curtis A. Suttle

**Affiliations:** 1 Department of Botany, University of British Columbia, Vancouver, BC V6T 1Z4, Canada; marlivlok@alumni.ubc.ca; 2 Emeritus Faculty, Australian National University, Canberra, ACT 2601, Australia; 3 Institute for the Oceans and Fisheries, University of British Columbia, Vancouver, BC V6T 1Z4, Canada; 4 Department of Earth, Ocean and Atmospheric Sciences, University of British Columbia, Vancouver, BC V6T 1Z4, Canada; 5 Department of Microbiology and Immunology, University of British Columbia, Vancouver, BC V6T 1Z3, Canada

**Keywords:** Charavirus, RNA viruses, capsid proteins, metagenomes, virome, phylogenetics

## Abstract

Charophyte algae, not chlorophyte algae, are the ancestors of ‘higher plants’; hence, viruses infecting charophytes may be related to those that first infected higher plants. Streamwaters from British Columbia, Canada, yielded single-stranded RNA metagenomes of Charavirus canadensis (CV-Can), that are similar in genomic architecture, length (9593 nt), nucleotide identity (63.4%), and encoded amino-acid sequence identity (53.0%) to those of Charavirus australis (CV-Aus). The sequences of their RNA-dependent RNA-polymerases (RdRp) resemble those found in benyviruses, their helicases those of hepaciviruses and hepegiviruses, and their coat-proteins (CP) those of tobamoviruses; all from the alphavirus/flavivirus branch of the ‘global RNA virome’. The 5’-terminus of the CV-Can genome, but not that of CV-Aus, is complete and encodes a methyltransferase domain. Comparisons of CP sequences suggests that Canadian and Australian charaviruses diverged 29–46 million years ago (mya); whereas, the CPs of charaviruses and tobamoviruses last shared a common ancestor 212 mya, and the RdRps of charaviruses and benyviruses 396 mya. CV-Can is sporadically abundant in low-nutrient freshwater rivers in British Columbia, where *Chara braunii*, a close relative of *C. australis*, occurs, and which may be its natural host. Charaviruses, like their hosts, are ancient and widely distributed, and thus provide a window to the viromes of early eukaryotes and, even, Archaea.

## 1. Introduction

Charophyte algae are the sister group of the chlorophyte (green) algae. Molecular genetics indicates that they gave rise to higher plants 450–500 mya [1,2]; hence it is sensible to search for viruses infecting extant charophytes as they may provide evidence of the virus lineages that infected the earliest higher plants. The discovery of sequences in metagenomic data from freshwaters in British Columbia, Canada that were homologous to those of a virus, we call Charavirus australis (CV-Aus), found in plants of *Chara australis* in rivers of south-eastern Australia [3,4] provided an opportunity to compare charaviruses and their relatives in space and time; in space because *C. australis* is found in south-east Asia and Australasia, but not in the Americas, and in time because they share two genes with the tobamoviruses, for which an age has recently been proposed [5], allowing a wider extrapolation of indicative dates.

CV-Aus was found in the 1970s in plants of *C. australis,* a large charophyte growing in the Murrumbidgee River near Canberra, Australia. Its virions were detected in sap from infected thalli by electron microscopy and serology, and stem cells of some infected thalli contained large paracrystals of virions formed by cytoplasmic streaming, and detected by polarized light microscopy and electron microscopy [3]. No similar virions were subsequently found in a limited number of samples of charophytes from other rivers in Australia, from rivers and lakes of central and southern England, nor from a worldwide collection of live charophytes kept by V.W. Proctor [4,6]. However, two short (862 and 587 bp) CV-Aus-like contigs were reported from metagenomic data of samples collected in the Finger Lakes (NY, USA) [7], confirming the presence of chara-like viruses outside of Australia.

CV-Aus has always appeared to be taxonomically anomalous. Its virions resemble those of Tobacco Mosaic Virus (TMV), with which it was originally grouped (International Committee in Taxonomy of Viruses, 3rd Report), in that they are straight helically-constructed tubes, 18 nm wide and with a similar pitch, but they are significantly longer; about 530 nm in length rather than 300 nm [3]. The CV-Aus genome is also commensurately longer than that of TMV; around 9 kb rather than the 6.4 kb, but was predicted to be >9.8 kb as its 5’terminus seemed incomplete [4]. The CV-Aus genome encoded a >227 kDa replicase that has helicase, protease, and RdRp domains, a 44 kDa helicase, a 38kDa movement protein, and an 18 kDa capsid protein (CP). The last has significant sequence similarity to the CPs of tobamoviruses, and hence the structural similarity of their virions, whereas the replicase shares predicted amino-acid similarity with those of beny- and hepeviruses, and the helicase with those of the helicases of hepaciviruses and hepegiviruses. These unusual gene sequence relationships make charaviruses a likely source of insights into the study of virus origins.

Here, we report on the assembly of RNA virus metagenomic data from freshwaters near Vancouver, British Columbia [8] that yielded the genome of a putative virus, which we call Charavirus canadensis (CV-Can), which closely resembles CV-Aus. Through comparative genome analysis, we explore the evolutionary, temporal, biological, and taxonomic relationships of these viruses, and use metagenomic analysis to infer the abundance and diversity of CV-Can in the environment. Our analyses indicate that plants of other species in the genus, *Chara*, are the likely host of CV-Can.

## 2. Materials and Methods

Viral RNA metagenomic sequences were downloaded from the NCBI Sequence Read Archive, BioProject accession PRJNA287840 [8]. These data were derived from samples collected from three streams in southwestern British Columbia over a 14-month period. A full account of the collection and subsequent generation of the metagenomic data are described in [8,9,10].

### 2.1. Metagenomic Assembly and Genomic Analysis

Reads from each library had primer sequences removed and quality trimmed (PHRED score of 20) using Trimmomatic v0.3 (http://www.usadellab.org/cms/?page=trimmomatic) [11]. Final datasets consisted of merged paired reads (PEAR v10, https://cme.h-its.org/exelixis/web/software/pear/, [12]) and singletons. The reads from each dataset were assembled separately using the de novo assembly algorithm in CLC Genomics Workbench version 7.5, with default settings (CLCBio, Cambridge, MA, USA). The contigs, as well as unmapped reads for each metagenomic dataset, were combined and re-assembled using the same parameters. Relative abundances were determined by mapping reads from each dataset to the final contigs, rarefied to the smallest sample size (158,376) using the phyloseq package [13] in R 3.2.2 (https://www.r-project.org/) [14] with 1000 iterations and normalized by the number of reads divided by the average contig size.

Genome nucleotide similarities were calculated using Bioedit [15] and visualized using the LAGAN global pairwise alignment program [16] as part of the mVISTA webserver [17,18]. Amino acid similarities and identities were determined using Bioedit [15] and the pairwise alignment algorithm of Geneious Prime 2019.0.4 (https://www.geneious.com). Related sequences in the Genbank database were identified using its online BLAST facilities (Jan–Oct 2017). Sequence alignments were tested for phylogenetic anomalies using RDP.v.4.95 (http://web.cbio.uct.ac.za/~darren/rdp.html) [19].

### 2.2. Phylogenetic Analysis

The charavirus and other sequences were sorted, checked, grouped, and translated and duplicates removed using MAFFT-v.7.313 (https://mafft.cbrc.jp/alignment/software/) [20], the Neighbor-Joining (NJ) facility in ClustalX [21], BioEdit-v.7.0.5, and the TranslatorX server [22] (http:// translatorx.co.uk) with its MAFFT option. Models for maximum likelihood (ML) analysis of the sequences or their encoded protein sequences were compared using MEGA-v.7.0.26 [23]. Phylogenetic trees were inferred using the ML method PhyML-v.3.0 (ML) [24] and statistical support for their topologies assessed using the Shimodara Hasegawa (SH) option [25]. ML trees were visualized and midpoint rooted using Figtree 1.4.2 (http://tree.bio.ed.ac.uk/software/figtree/) and drawn using a commercial computer illustration package. The evolutionary divergence of the charaviruses and tobamoviruses was estimated from the ML trees of protein sequences using Patristic [26] and MS Excel; the dates of nodes in trees were compared using the ratios of the mean pairwise patristic distances of all sequences (i.e., tips) connected through individual nodes; the date of one node in a tree provided an estimate of others in the same tree.

### 2.3. K-mer Analysis

K-mer counts of the RdRp and capsid coding sequences were determined using the biostrings package [27] in R 3.2.2 [14] using Rstudio 0.99.489 [28], and were compared by correspondence analysis [29,30] in the vegan [31] and vegan3d [32] packages.

### 2.4. Single Nucleotide Variant Analysis

Metagenomic reads were mapped to the genome using the CLC Genomics Workbench v7.5 with a stringency of identity and read overlap of 90%. Analysis of single nucleotide variation (SNV) was done using the Quality-based Variant Detection tool in CLC Genomic Workbench v7.5 (minimum read count of two) and visualized using R 3.2.2 [14] and the ggplot2 package [33].

## 3. Results

### 3.1. The CV-Can Metagenome

A single-stranded RNA metagenome (CV-Can; Accession Code MK521928) was assembled from multiple lotic freshwater samples collected from southwestern British Columbia. The genome was 9593 nt (2.99 megadaltons) in length with a G + C content of 46.0%, but only 15.1% guanine. A BLAST search of Genbank found the sequence matched most closely, throughout most of its length, that of CV-Aus [4] (Accession Code JF 824737). The CV-Aus genome is only 9065 nt (2.78 daltons) in length with a G + C content of 45.0%, and like that of CV-Can, had a noticeably small (14.7%) guanine content.

**Figure 1:**
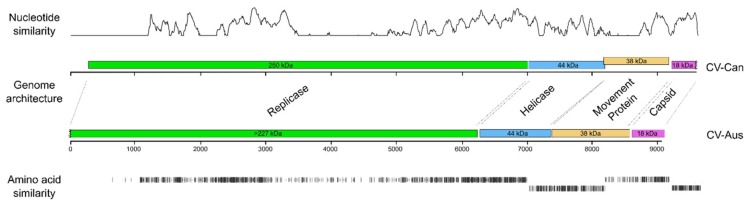
Comparison of two Charavirus genomes; gene map and similarities of nucleotide sequences and predicted amino acids. The nucleotide similarity axis positively correlated with the similarity score. Predicted weight of putative proteins are indicated in kilo Daltons (kDa); identical amino acids in black, similar amino acids in grey and in line with the open reading frame (ORF) of Charavirus canadensis (CV-Can).

The CV-Can sequence has four non-overlapping open reading frames (ORFs) that match in size and order with those reported for CV-Aus (Figure 1), except that the longest ORF, which encodes a replicase (Rep) and is adjacent to its 5′ terminus, is 6732 nt long (69% of the metagenome); whereas, that of CV-Aus is only around 6215 nt. This difference is because of an extra region at the 5′ terminus of the CV-Can sequence that encodes a putative viral methyltransferase (v-Mtase) domain, which was predicted to be missing from the incomplete 5′-terminus of the CV-Aus sequence [4]. The three smaller ORFs are predicted to encode a helicase (Hel), a possible movement protein (MP), and a virion coat protein (CP). The concatenated ORFs of CV-Can and CV-Aus had a mean nucleotide sequence identity of 63.4%, and predicted mean amino-acid sequence identity of 53.0%. The predicted amino-acid sequences of the Charavirus Rep proteins were 53.0% identical, the Hel proteins 53.1% identical, the MPs proteins only 44.4% identical, and the CPs 66.7% identical; the CPs were most conserved and the MPs least, as found with many similar viruses.

### 3.2. Predicted Gene Products of CV-Can

#### 3.2.1. Replicase (nt 276 to 7007)

In a BLASTn search using the longest ORF of the CV-Can genome against the Genbank non-redundant (nr) database, the only significant sequence match (p 2e^−141^) was to the putative replicase in CV-Aus. Translated, the ORF is predicted to code for a protein of 250 kDa. BLASTp and Pfam reveal that adjacent to the N-terminus is a viral methyl-transferase region (v-MTase); centrally, there is significant similarity to RNA helicase-1 (v-Hel)(PF01443), and the C-terminus encodes an RNA dependent RNA polymerase-2 (RdRp-2)(PF00978) region. These motifs are ‘hallmark domains’ of single-stranded RNA (ss-RNA) viral genomes of the Alphavirus or ‘Sindbis-like’ superfamily [34,35,36]. The translated sequences for these individual motifs of the replicase were used to search the Genbank nr-database using BLASTp.

The v-MTase region of the replicase (amino-acid residues 1–500) most closely matched the same region of the CV-Aus sequence. It also matched significantly (SH support >0.84) v-MTases encoded by benyviruses and related viruses (i.e., beet necrotic yellow vein, beet soil-borne mosaic, rice stripe necrosis, burdock mottle, and Mangifera indica latent viruses [37,38,39], and also Hubei Beny-like virus 1 [40] and Agaricus bisporus virus 8 (KY357493) [41].

The v-RNA Hel-1 region (residues 710–945) matched most closely helicases encoded by the same region in benyvirus genomes and, more distantly, helicases from Hubei Benyi-like virus 1 and Hubei Hepe-like virus 3 [40], and Lentinula edodes ssRNA mycovirus (AB647256) [42].

**Figure 2:**
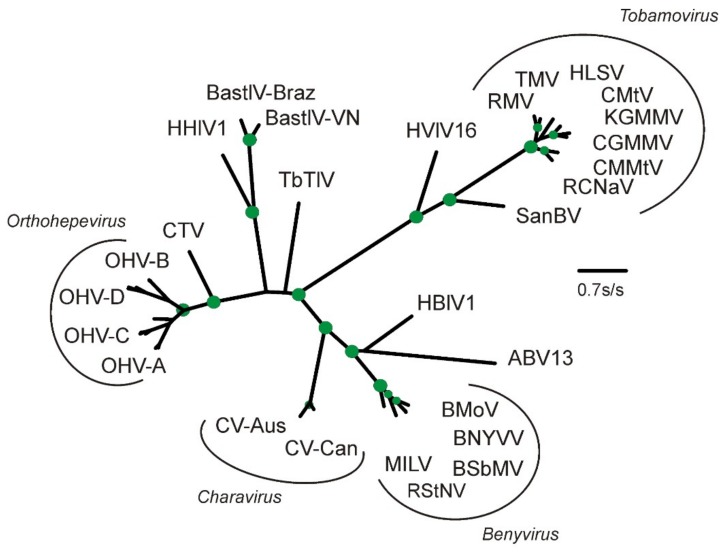
Maximum-likelihood phylogeny of the amino acid sequences of the RdRp-2 regions of the replicase proteins of the charaviruses, benyviruses, and selected tobamoviruses and relatives. Acronyms: ABV13, Agaricus bisporus virus 13 (AQM49942); BastlV-VN, Bastrovirus-like_virus-VietNam Bat (YP_009333174); BastV-Braz, Bastrovirus Brazil/sewage (ASM79505); BMoV, Burdock mottle virus (YP_008219063); BNYVV, Beet necrotic yellow vein virus (NP_612615); BSbMV, Beet soil-borne mosaic virus (NP_612601); CGMMV, Cucumber green mottle mosaic virus (NP_044577); CMMtV, Cactus mild mottle virus (YP_002455590); CTV, Cutthroat trout piscihepevirus (YP_004464917); CuMtV, Cucumber mottle virus (YP_908760); CV-Aus, Charavirus australis (AEJ33768); CV-Can, Charavirus canadensis (MK521928); HBlV1, Hubei Beny-like virus 1 (APG77690); HHlV1, Hubei hepe-like virus 1 (YP_009336840); HLSV, Hibiscus latent Singapore virus (YP_719997); HVlV16, Hubei virga-like virus 16 (YP_009336677); KGMMV, Kyuri green mottle mosaic virus (YP_908760); MILV, Mangifera indica latent virus (AMQ23297); OHV-A, Orthohepevirus A(ABB88699, AGE83293, AGE83340, AGT38396, ANW09725, BAE86910); OHV-B, Orthohepevirus B (AEX93357, CAQ16023, YP_009001465; OHV-C, Orthohepevirus C (ADB96199,AFL69932, ANJ02843, BAO47898, BAT70058); OHV-D, Orthohepevirus D (AIF74285, YP_006576507); RCNaV, Rattail cactus necrosis-associated virus (YP_0044936166); RMV, Ribgrass mosaic virus (YP_005476600); RStNV, Rice stripe necrosis virus (ABU94739); SanBV, San Bernardo virus (AQM55436); TbTlV, Tick borne tetravirus-like virus (AII01815); TMV, Tobacco mosaic virus (NP597746). The green discs mark nodes with >0.9 SH support; two thirds of the nodes in the Orthohepevirus cluster have >0.9 SH support, but, for clarity, are not marked.

The RdRp-2 region (residues 1830–2220) significantly matched more than 500 viral RdRp sequences in Genbank. The closest matches are again those of the benyviruses (Figure 2), as well as Agaricus bisporus virus 13 (KY357498) [41] and Hubei Beny-like virus 1 [40]. The basal sister group is dominated by a crown group of RdRp proteins from avian and mammal hepeviruses. Thus, the CV-Can replicase sequence matches the replicase sequence in CV-Aus, as well as many other more distantly related sequences in Genbank from ‘environmental samples’ rather than cultured or isolated virions.

#### 3.2.2. Helicase (nt 7014–8192)

The putative helicase encoded by this ORF is predicted to be a 44 kDa protein that closely matches that from CV-Aus based on a BLASTp analysis, and is more distantly related to helicase sequences of hepaciviruses and hepegiviruses of animals [4]. It includes a motif (residues 25–170) of the DEAD-like (DEXc) helicase superfamily, a diverse family of proteins involved in ATP-dependent RNA or DNA unwinding [43].

#### 3.2.3. Possible Movement Protein (nt 8197–9117)

The translated sequence of this ORF encodes a putative protein with a predicted size of 38 kD. It is homologous to an ORF in the same genomic position in CV-Aus, but they had the smallest sequence similarity of all pairwise comparisons of the ORFs of the two viruses. The encoded proteins matched no others in the Blastp or Pfam database searches. The properties of the CV-Aus protein were discussed in detail in the report of the CV-Aus genome [4]. Their position in the genome is typical of the movement proteins of many of the plant-infecting virgaviruses [44,45].

#### 3.2.4. Coat protein (CP) (nt 9197–9631)

**Figure 3:**
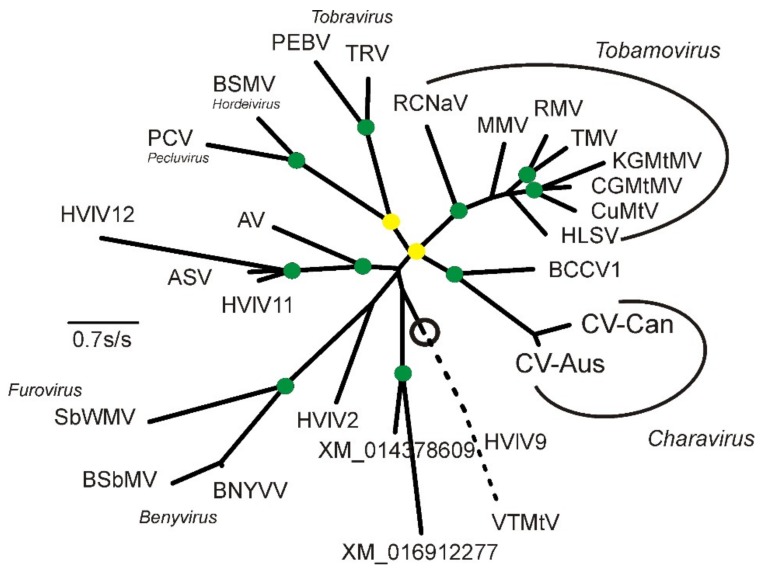
Maximum-likelihood phylogeny of the amino acid sequences of the CPs, and CP-like proteins, of the charaviruses and relatives. These include: AV, Abisko virus (NC_035470); ASV, *Adelphocoris suturalis* virus (NC_032728); BCCV1, Beihai *Charybdis* crab virus 1 (NC_032449); BNYVV, Beet necrotic yellow vein virus (NC_003515); BSbMV, Beet soil-borne mosaic virus, (NC_003503); BSMV. Barley stripe mosaic virus (NC_003481); CGMtMV, Cucumber green mottle mosaic virus (NC_001801); CuMtV, Cucumber mottle virus (NC_008614); CV-Aus, Charavirus australis (JF824737); CV-Can, Charavirus canadensis (MK521928); HLSV, Hibiscus latent Singapore virus (NC_008310); HVlV2; Hubei virga-like virus 2 (NC_033158); HVlV9, Hubei virga-like virus 9 (NC_032765); HVlV11, Hubei virga-like virus 11 (NC_033082); HVlV12, Hubei virga-like virus 12 (NC_033269); KGMtMV, Kyuri green mottle mosaic virus (NC_003610); MMV, Maracuja mosaic virus (NC_008716); PCV, Peanut clump virus (NC_003668); PEBV, Pea early browning virus (NC_001368); RCNaV, Rattail cactus necrosis-associated virus (NC_016442); RMV, Ribgrass mosaic virus (NC_002792); SbWMV, Soil-borne wheat mosaic virus (NC_002042); TMV, Tobacco mosaic virus (NC_001367); TRV, Tobacco rattle virus (NC_003811); VTMtV, Velvet tobacco mottle virus (NC_014509); XM_014378609, a gene from *Apis mellifera*; XM_016912277, a gene from *Trichogramma pretiosum*. The circle marks the midpoint of the phylogeny, and the broken line is the branch to the outgroup of HVlV9 and VTMtV drawn only at 50% of its true length. The green discs mark nodes with >0.9 SH support and the yellow discs those with >0.8 < 0.9 SH support.

This ORF is predicted to encode an 18 kDa protein with greatest identity to the comparable sequence in CV-Aus, indicating that they are the most evolutionarily conserved (Figure 3), particularly for amino-acid residues 1–132. We were unable to identify ‘read contigs’ with alternative 3-termini matching the CV-Aus sequence, suggesting that the CV-Can metagenome is missing the 3′-terminal 22 codons of the CP gene homologous to those of CV-Aus.

The highly conserved 132 amino-acid sequence was used in successive BLASTp searches of the Genbank database. In addition to matching several tobamovirus CPs, it matched several tobamo CP-like proteins from invertebrates identified in metagenomic studies. Based on an ML taxonomy (SH support 0.94) (Figure 3), the protein most similar to the charavirus CPs was a predicted protein of the Beihai *Charabydis* crab virus 1 (BCCV1; NC_032449) [40], which was assembled from metagenomic data.

### 3.3. Divergence of CV-Aus and CV-Can

Molecular clock analysis of the CP was used to estimate the divergence times between charaviruses and tobamoviruses based on the proposal that tobamoviruses are as old as their angiosperm hosts [5], which are therefore estimated to have diverged around 130 million years ago [46,47]. Divergence dates were estimated from the maximum-likelihood (ML) tree of the RdRps and CPs of the charaviruses and tobamoviruses (Figure 2 and Figure 3) assuming that patristic distances in the tree are linearly related to evolutionary time. For example, the mean patristic distance between the CP of rattail cactus necrosis-associated virus and the other seven tobamoviruses (i.e., the basal node of the tobamoviruses) is 2.123 +/− 0.193 amino-acid substitutions/site (aas/s), whereas the distance between the two charavirus CPs is 0.468 aas/s. These distances suggest that the two charavirus CPs diverged 28.7 million years ago. Likewise, the mean pairwise patristic distance between the two charavirus CPs and each of the eight tobamovirus CPs is 3.467 +/− 0.239 aas/s., suggesting that the charavirus and tobamovirus CP genes diverged ~212 million years ago. The aligned CP sequences had several indel-rich regions, resulting in an average of 258.8 gaps in each sequence of the 447 sites, but when the sequences were partitioned to remove the sites that contributed the most gaps, leaving sequences of 165 aa with an average of 14.8 gaps/sequence, they gave almost identical date estimates. Similar calculations were used to date the RdRps (Supplementary Table S1).

**Figure 4:**
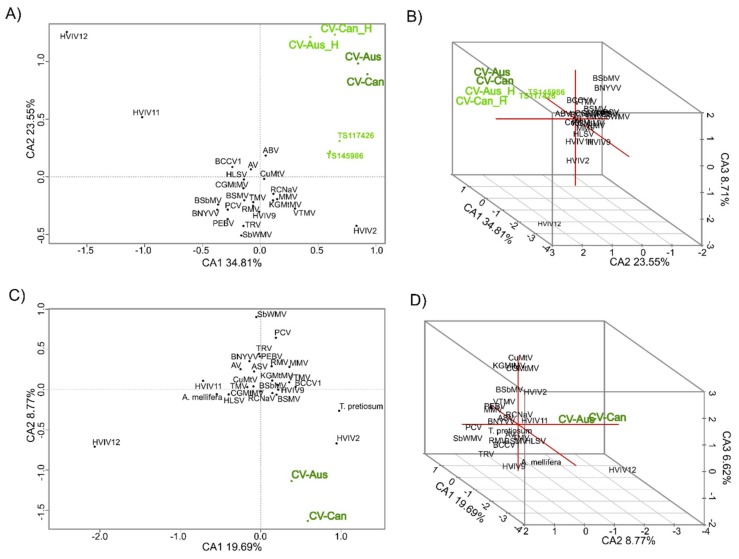
Correspondence analysis of tetra-nucleotide patterns of charaviruses and other sequence related viruses. Patterns were obtained for both RdRp sequences (**A**,**B**) used in Figure 1, and capsid gene sequences (**C**,**D**) from Figure 2. The separated helicase sequences of the two charaviruses (CV-Aus_H and CV-Can_H) were included to compare with the helicase sequences reported by [7], but can be seen to be distinct.

**Figure 5:**
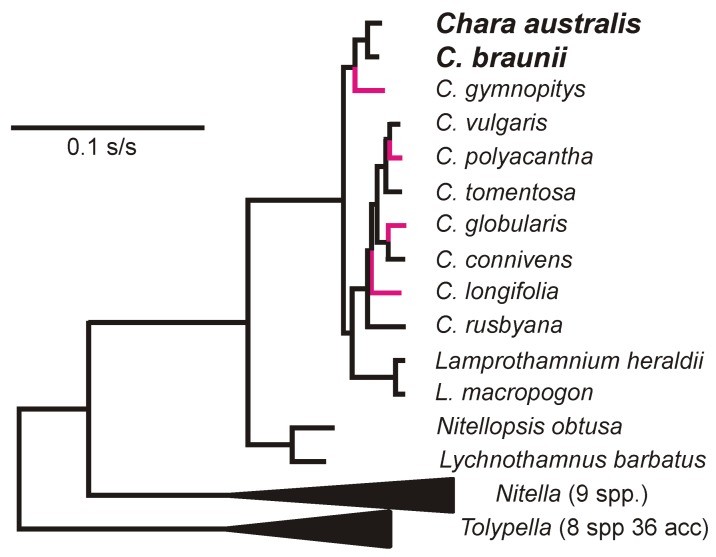
Molecular phylogeny of charophytes as determined by Perez et al. [49], with four additional species (red branches) extrapolated from the data of Sakayama et al. [48].

CV-Aus and CV-Can genes have k-mer patterns, especially those of tetra-nucleotides, that are distinct and place the charavirus RdRp and CP genes together and separate from the genes of other sequence-related viruses (Figure 4). Also, the k-mer patterns of the two charavirus helicases were more similar to those of their RdRps than the two helicase-like sequences (TS117426 and TS145986) obtained from the freshwater lakes of New York State. These patterns suggest (see Discussion) that CV-Can and CV-Aus infect closely related hosts, charophytes, but those from New York State may have a different host. To identify more specifically the likely host of CV-Can, we melded two molecular phylogenies of charophytes, one based on the *rbcL* gene [48] and the other the combined *atpB*, *psbC*, and *rbcL* genes [49]. This showed that *C. braunii*, which has been reported from British Columbia, is the sister taxon of *C. australis* (Figure 5), a relationship that has 93% and 100% bootstrap support in the separate taxonomies.

### 3.4. CV-Can in British Columbia

**Figure 6:**
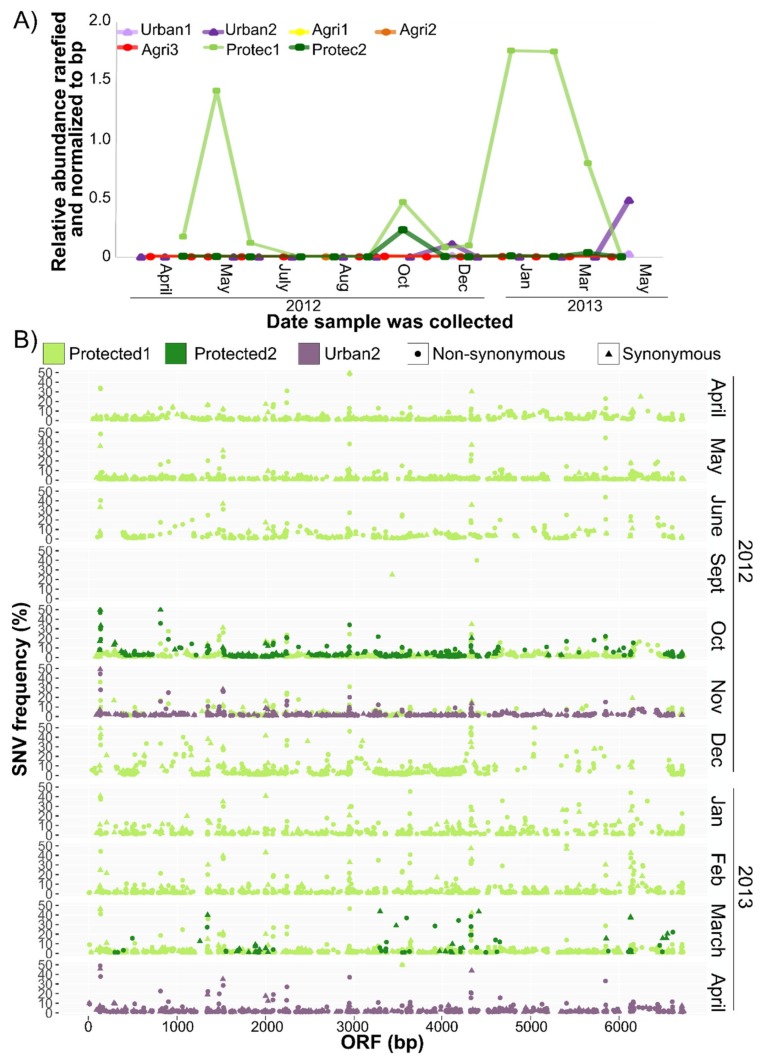
CV-Can contigs were abundant and diverse in freshwater streams. Relative abundances calculated from rarefied read counts and normalized by contig size (**A**) suggest that CV-Can was most abundant at the first protected site (Prist1) compared to the urban and agricultural (Agri) sites. Single nucleotide variant (SNV) analysis (**B**) of the replicase gene at the two protected and one urban site indicate much genetic variation within the CV-Can population.

The CV-Can metagenome was abundant in water sampled from a shallow river in a forested, protected watershed (Protec1), downstream from a reservoir fed from Protec1 after passing through an 8.8 km pipe (Protec2), and from a shallow stream approximately 1 km from a residential neighbourhood (Urban2) (Figure 6A). Relative abundances fluctuated throughout the year, with Protec1 being the most distinct. CV-Can was not detected in samples collected from three locations along a stream near agricultural sites.

Occasional ‘single nucleotide variants’ (SNVs) of the consensus CV-Can sequence were detected (Figure 6B). The SNVs were detected in all contigs (Supplementary Figure S1), with the replicase having the most consistent coverage both across the sequence length and samples. Interestingly, the majority of the SNVs encoded non-synonymous changes.

## 4. Discussion

In this study, we used metagenomic data collected from freshwater habitats in southwestern British Columbia to assemble the metagenome of a previously unknown single-stranded (ss) RNA virus (CV-Can) with significant sequence similarity to a virus (CV-Aus) isolated from *Chara australis* growing in Australia. These data, together with the report of metagenomic fragments of a third possible Charavirus population from lakes in New York State [7], indicates that charaviruses may be more widespread than previously thought.

All the significant features of CV-Can are shared with those reported for the genome of CV-Aus [3,4,6]. The compositions, lengths, number, and sizes of ORFs and their predicted proteins are similar, but they differ significantly at the individual codon level. The CPs were most similar (71.3% nt, 66.7% aa identity), and the MPs least (62.1% nt, 44.4% aa identity). These identities are equivalent to those of different tobamoviruses; comparisons of 48 tobamoviruses representing all species of the genus found their CPs had modal identities of ~57% nt (range 53%–62%) and ~36–42% aa (range 26%–52%); whereas, the CPs of the closely related, but distinct, tobacco mosaic and tomato mosaic viruses have ~75% nt and 84% aas identity. Thus, we conclude that CV-Can and CV-Aus represent distinct species of a new proposed genus, *Charavirus*.

Subak-Sharpe et al. [50] and Hay and Subak-Sharpe [51] first showed that the nearest-neighbour nucleotide composition of the genomes of small viruses was closely similar to those of their hosts, probably reflecting their adaptation to the transfer RNA population of their hosts. Moreover, Kapoor et al. [52] showed that the mono and dinucleotide composition of RNA virus genomes was a useful predictor of their hosts’ kingdom or phylum, probably for the same reason. The similarity between virus and host k-mer patterns have further been validated in virus-host systems [53]. We analyzed the k-mer patterns (from di-nucleotide to septa-nucleotides) of the ORFs of charaviruses and their phylogenetic relatives, including two published charavirus-like helicase sequences [7]. Here, we report that the k-mer patterns of the RdRp and CP genes of the charaviruses were more similar to one another than to those of other phylogenetically related viruses, suggesting that they have related hosts, and hence that the natural host of CV-Can is probably a charophyte.

The consistent linkage of the charavirus CPs to a homolog isolated from a swimming crab, Beihai Charybdis crab virus 1, is of interest. This virus is possibly a contaminant, rather than a virus of Charybdis crabs, as, although these crabs are opportunistic carnivores, they do eat algae [54], and although most charophytes inhabit freshwater, some also live in brackish water [55], where they might be eaten by crabs. However, in the k-mer analyses, the BCCV1 CP did not group with the CV CPs, indicating that its host is probably not a charophyte.

The natural host of CV-Aus, *C. australis*, has only been recorded from rivers in Australia, India, Malaysia, New Caledonia, New Zealand, and Taiwan [56], but not the Americas. However, two molecular genetic studies of various charophytes, including *C. australis*, were congruent and clarified their phylogeny. Sakayama et al. [48] used rbcL sequences from 10 *Chara* spp. and 17 other charophytes, whereas Pérez et al. [49] combined atpB, psbC, and rbcL sequences from 6 *Chara* spp. and 20 other charophytes. Both phylogenies found *C. braunii* to be the sister taxon to *C. australis* (Figure 5). In contrast to *C. australis*, *C. braunii* is cosmopolitan [56]. It is one of 27 Chara species (84 charophytes) found in North America [57] and one of 13 Chara species in British Columbia, with *C. braunii* recorded there at three sites (Henry Mann; personal communication). There are many morphotypes of plants in the species of *C. braunii* [56], and mating experiments [58] showed that not all populations of *C. braunii* were compatible, and none were compatible with one *C. australis* population. Although it is possible that *C. braunii* is the natural host of CV-Can, we have not confirmed this.

The relative abundance of CV-Can sequences in rivers of British Columbia varied over a 14-month period (Figure 6A), and many non-synonymous SNVs were detected in the CV-Can population (Figure 5B). Similar variation was found in the New York metagenomic sequences [7] (Ian Hewson, personal communication). The dominance of non-synonymous SNVs suggests that the British Columbia rivers had more than one distinct CV-Can population; high levels of non-synonymous mutations were reported in tobamoviruses when the viruses were passaged in a new host [59]. While there is no way of investigating these possibilities with the present data, the majority of SNVs were infrequent, suggesting that they have not been fixed within the population, and perhaps the CV-Can population of British Columbian rivers is, over the long term, a mega viral quasispecies [60].

The longest assembled sequence of the third possible record of a Charavirus from New York State (TS145986) was 862 nt, and it had around 70% nucleotide identity (80% amino-acid identity) with the homologous region from both CV-Can and CV-Aus. Thus, there was no correlation between the sequence differences of this region and the geographic distances between collection sites (~4,000 to ~16,000 km), suggesting that the New York State sequence was from a distinct charavirus lineage, not from the CV-Can/CV-Aus lineage.

The detection of CV-Can at some locations, but not others, likely reflects the distribution and abundance of host populations. *Chara* spp. are found in slow flowing, frequently shallow lakes and rivers or streams that are nutrient-poor [61,62], where they thrive, in part, due to the co-occurrence of nitrogen-fixing epiphytic cyanobacteria [63]. The protected site, where CV-Can was abundant (Figure 6A), is a low nitrogen environment, and is distinct from the agricultural sites where inorganic nitrogen concentrations are higher [8,64]. The presence of CV-Can in the protected watershed suggests that it could be used as an indicator species of low nitrogen and low turbidity freshwater environments in British Columbia [64]. Similar studies could not be done at the original site where CV-Aus was found as *C. australis* plants are now rare as a result of drought, siltation, and fertilizer use over the past four decades [4].

The phylogenies of the individual Charavirus genes we report here are completely congruent with the recently published phylogeny of the ‘global RNA virome’ [65]. The charavirus RdRp, helicase, and CP genes are related individually to genes of different viruses in ‘branch 3’ of the Wolf et al.’s phylogeny, which includes the alphavirus and flavivirus supergroups [43,66,67]; the RdRp and CP genes belong to different lineages of the alphaviruses, and the helicases to those of flaviviruses. However, in all instances, the charavirus protein is a sister (basal) to those of viruses of extant related groups, indicating an ancestral relationship to them all, rather than recent inter-lineage recombination events.

**Figure 7:**
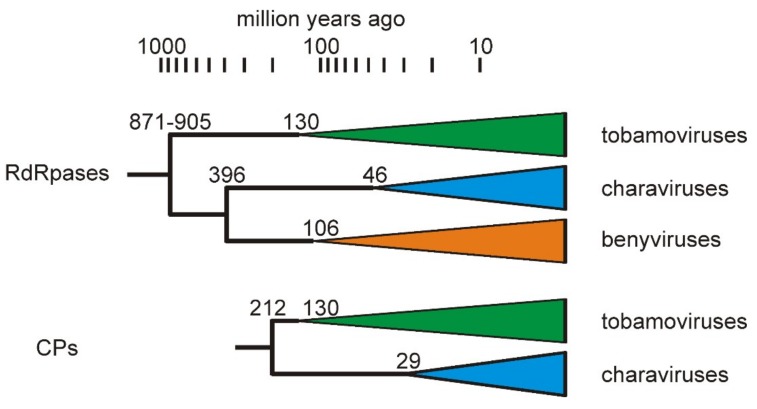
Summary of the estimates of gene divergence dates (million years before present) for the charavirus, tobamovirus, and benyvirus RdRp and CP proteins. The coloured triangles represent monophyletic clusters (i.e., two or more homologs) of the proteins found in extant viruses, and their left hand tip represents the earliest likely age of each extant virus cluster.

A statistically significant correlation has been found between the phylogeny of most tobamoviruses and their primary eudicotyledonous hosts [5], confirming an earlier estimate of their age based on protein evolution rates [68], and indicating that tobamoviruses probably co-evolved with their hosts and are thus of a similar age, which is now estimated to be around 130 million years [69]. This date can then be used to extrapolate from the two tobamovirus genes, RdRps and CPs, to other nodes in their respective gene phylogenies by comparing patristic distances that pass through those nodes (Supplementary Table S1). Caution must, of course, be used in interpreting these estimates (Figure 7) as more than indicative, given the analytical uncertainties generated by model choice, protein size, etc. The divergence of the two charaviruses was estimated to be 46 mya (RdRps) and 29 mya (CPs), which places it in the same time period as the breakup of Gondwana, and the final separation of Australia from Antarctica, and hence the Americas, ~30 mya. This divergence correlates with the known distribution of *C. australis*, which is confined to lands that once formed East Gondwana [70,71], whereas *C. braunii* is cosmopolitan. The earlier divergence dates of the RdRps and CP genes (200–400 mya) are also feasible given that the earliest unequivocal fossils, gyrogonites (oogonia) of charophytes, are from early Devonian rocks, ~420mya [72]. An additional biochemical clue of the likely deep relationship between charophytes and viruses is that charophytes, but not chlorophytes (green algae) or rhodophytes (red algae), have R-genes encoding ‘nucleotide binding site-leucine rich repeat’ proteins [72], which modulate the response of higher plants to pathogens, such as TMV [73], enabling a ‘gene-for-gene’ modus vivendi [74].

Branch lengths of the ‘global RNA virome’ phylogeny [65] suggest that the earliest embodiments of the alphavirus/flavivirus RdRp genes existed more than one billion years ago, but the origins of the tobamovirus/charavirus CP gene are less certain. The structure of the TMV monomer is known in great detail [75]. It is a distinctive right-handed anti-parallel four helix bundle [75], that is uncommon among viruses of higher plants and animals [65] and probably confined to virions of viruses of the *Virgaviridae*, all of which have straight tubular virions. Similar helical bundle proteins, both left and right handed, seem to occur only in the viruses of Archaea [76], and may be the source of the virgavirid/charavirus CP gene.

The ancestors of members of the contemporary genus, *Chara*, are thought to be the closest extant relatives of algae that gave rise to higher plants. Consequently, charaviruses, which have clear evolutionary ties to tobamoviruses that infect higher plants, provide a window into the shared evolutionary history of these two groups of viruses. These observations suggest that tobamoviruses originated in aquatic environments and transitioned alongside their hosts to the terrestrial realm. Our results establish that charaviruses are a taxonomically distinct group with a distribution that includes Australia and North America. Moreover, analysis of viral metagenomic data imply that charaviruses occur as large genetically diverse populations that can be significant contributors to RNA virus metagenomic data in freshwater habitats. These results imply that charaviruses are likely important members of freshwater viral communities across the world.

## Supplementary Materials

The following are available online at https://www.mdpi.com/1999-4915/11/3/299/s1, Figure S1: Single nucleotide variants in the helicase (A), movement (B) and capsid (C) proteins of the CV-Can genome in protected and urban watersheds, Table S1: Mean Pairwise Patristic Distances of Selected Nodes.
