# Supplementary material for: Metagenomes of a Freshwater Charavirus from British Columbia Provide a Window into Ancient Lineages of Viruses
Source: Viruses. 2019 Mar 25;11(3):299. doi: 10.3390/v11030299 (PMC6466400; doi:10.3390/v11030299)
Supplement: Supplementary file 1 [file viruses-11-00299-s001.pdf]

Supplementary Table 1 – Mean Pairwise Patristic Distances of Selected Nodes.

RdRps	Viruses compared	Number of comparisons	Mean (s/s)	Stdev	Mya
	Chara x Charaviruses	1x1	0.3495		46.5
	Tobamo x Tobamoviruses	2x6	0.9780	0.0612	130
	Beny x Benyviruses	1x4	0.7970	0.0312	105.9
	Chara x Benyviruses	2x5	2.9823	0.1172	396.4
	Chara x Tobamoviruses	2x8	6.5583	0.1031	871.7
	Beny x Tobamoviruses	5x8	6.8087	0.1963	905.0
CPs					
	Chara x Charaviruses	1x1	0.4684		28.7
	Tobamo x Tobamoviruses	1x7	2.1217	0.1926	130
	Tobamo x Charaviruses	2x8	3.4671	0.2394	212.4

Distances for the CPs were derived from the taxonomy shown in Figures 2 and 3.

Supplementary figure 1: Single nucleotide variants in the helicase (A), movement (B) and capsid (C) proteins of the CV-Can genome in protected and urban watersheds.

A)

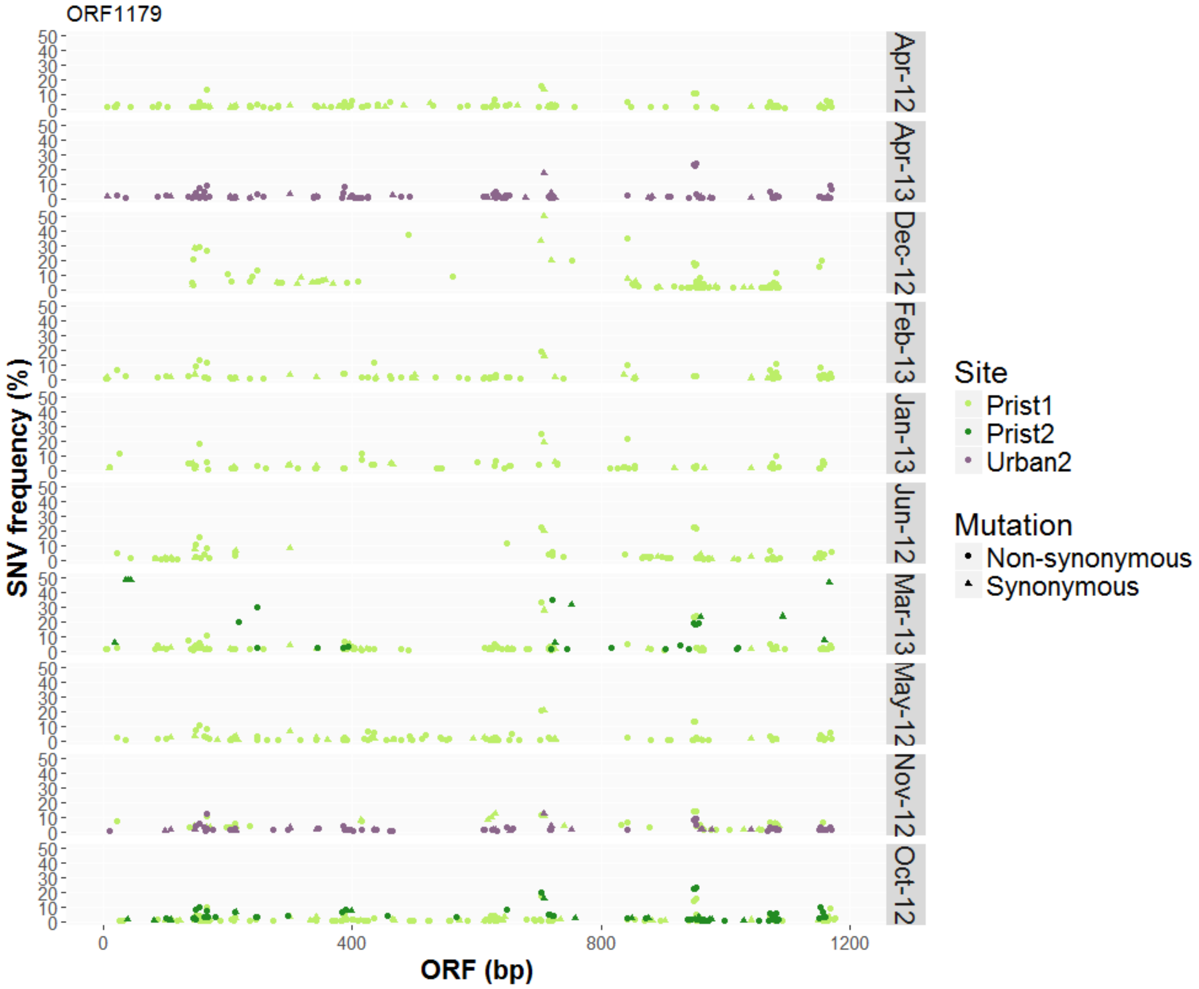

B)

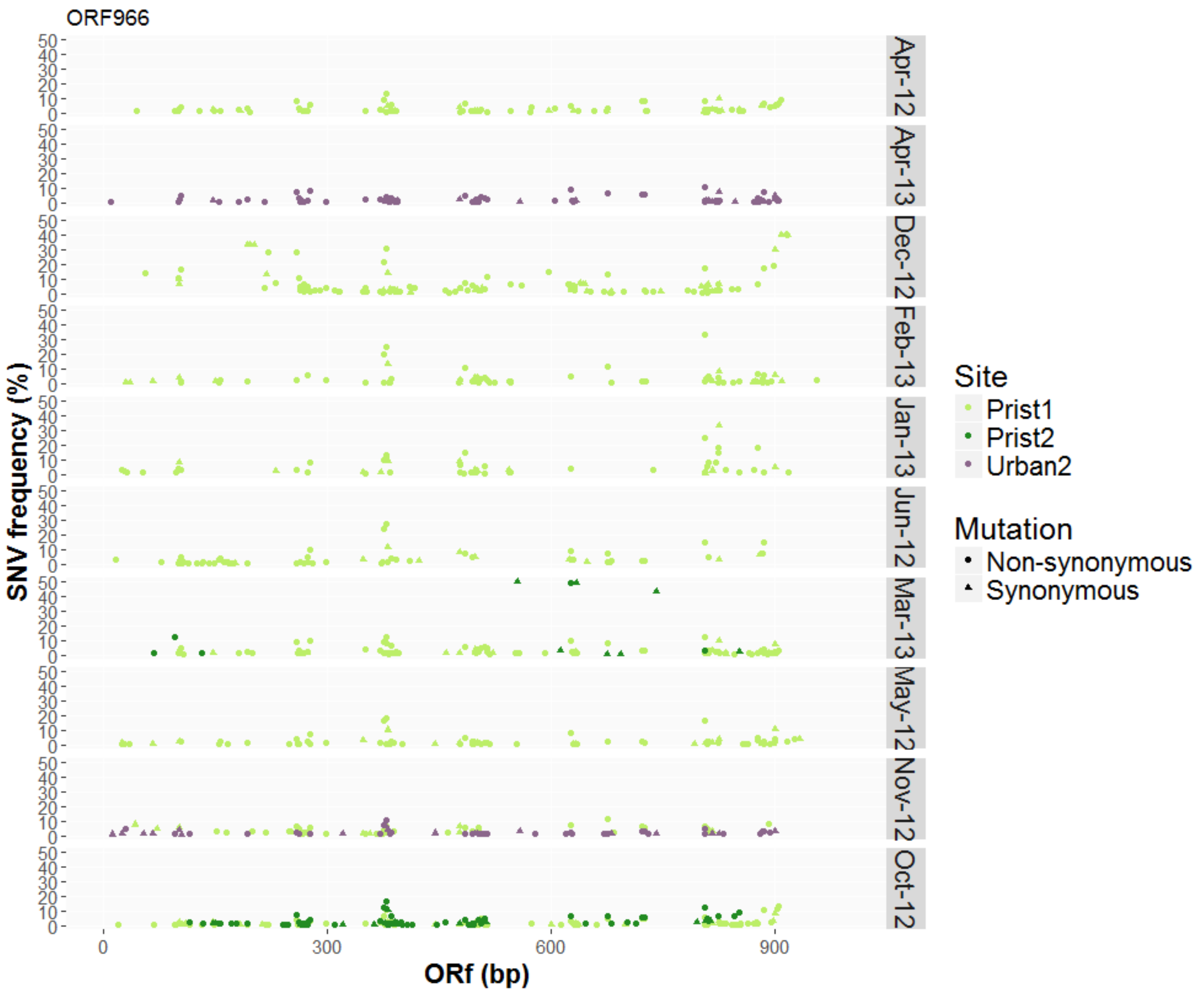
ORF966
SNV frequency (%)
ORf (bp)
Apr-12
Apr-13
Dec-12
Feb-13
Jan-13
Jun-12
Mar-13
May-12
Nov-12
Oct-12
Site
Prist1
Prist2
Urban2
Mutation
Non-synonymous
Synonymous

C)

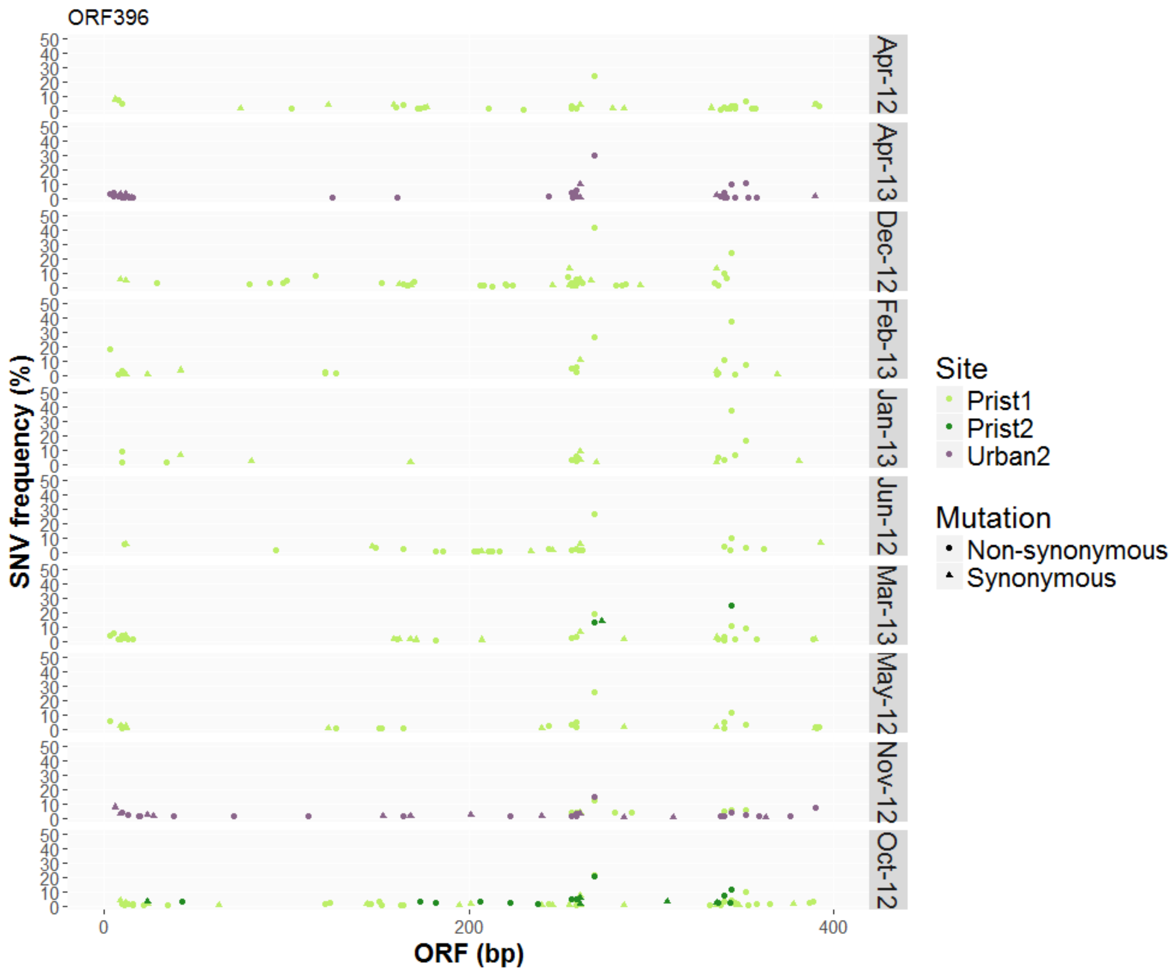
ORF396
SNV frequency (%)
ORF (bp)
Apr-12
Apr-13
Dec-12
Feb-13
Jan-13
Jun-12
Mar-13
May-12
Nov-12
Oct-12
Site
Prist1
Prist2
Urban2
Mutation
Non-synonymous
Synonymous
